# Pressure assisted enhancement in superconducting properties of Fe substituted NbSe2 single crystal

**DOI:** 10.1038/s41598-018-19636-z

**Published:** 2018-01-19

**Authors:** Manikandan Krishnan, Rukshana Pervin, Kalai Selvan Ganesan, Kannan Murugesan, Govindaraj Lingannan, Akshay Kumar Verma, Parasharam M. Shirage, Arumugam Sonachalam

**Affiliations:** 1 Centre of High Pressure Research, School of Physics, Bharathidasan University, Tiruchirappalli, 620024 India; 2 Discipline of Metallurgy Engineering and Materials Science & Physics, Indian Institute of Technology Indore, Simrol Campus, Khandwa road, Indore, 453552 India; 3 Department of Physics, University of Alabama at Birmingham, Birmingham, AL 35294 USA

## Abstract

The impact of hydrostatic pressure (P) up to 1 GPa on *T*_*c*_, *J*_*c*_ and the nature of the pinning mechanism in Fe_x_NbSe_2_ single crystals have been investigated within the framework of the collective theory. We found that the pressure can induce a transition from the regime where pinning is controlled by spatial variation in the critical transition temperature (*δT*_*c*_) to the regime controlled by spatial variation in the mean free path (*δℓ*). Furthermore, *T*_*c*_ and low field *J*_*c*_ are slightly induced, although the *J*_*c*_ drops more rapidly at high fields than at ambient P. The pressure effect enhances the anisotropy and reduces the coherence length, resulting in weak interaction of the vortex cores with the pinning centers. Moreover, the P can induce the density of states, which, in turn, leads to enhance in *T*_*c*_ with increasing P. P enhances the *T*_*c*_ with the rates of *dT*_*c*_*/dP* of 0.86, 1.35 and 1.47 K/GPa for Fe_x_NbSe_2_, respectively. The magnetization data are used to establish a vortex phase diagram. The nature of the vortices has been determined from the scaling behaviour of the pinning force density extracted from the *J*_*c*_*–H* isotherms and demonstrates the point pinning mechanism.

## Introduction

Technological applicability of superconductors is determined by several parameters such as high critical current density (*J*_*c*_), upper critical field (*H*_*c2*_), irreversibility field (*H*_*irr*_), strong magnetic flux pinning, high superconducting transition temperature (*T*_*c*_), small anisotropy and the capacity to sustain high critical current densities at raised temperatures^1–8^. Despite of high transition temperature (*T*_*c*_) in copper oxide based superconductors (with upper critical field in the excess of 150 Tesla), practically the entire superconducting magnet industry centers on liquid helium cooled niobium based superconductors. This is because the in-field critical current density, that is the maximum current density before the onset of diffusion at a given field, is rigorously suppressed in high *T*_*c*_ materials. Particularly perturbing is the presence of resistive behavior at extremely small current densities at all finite temperatures^9^. This happens on account of thermally activated hopping of vortex bundles across the pinning sites even if the Lorentz force due to the transport current is much smaller compared to the pinning force at the defect sites. In this respect the two band layered hexagonal compound dichalcogenide NbSe_2_ which undergoes the superconducting transition temperature (*T*_*c*_)~ is 7.2 K^1^, charge density wave (CDW) transition at T_CDW_ of about 30 K^2^ and the upper critical fields for H along and perpendicular to the hexagonal c-axis are *H*_*c2*_^*c*^ = 40 *kOe* and *H*_*c2*_^*ab*^ = 140 *kOe*, respectively^1^. The interplay between charge density wave order, i.e. a static modulation of the electronic density close to the Fermi level, and superconductivity has attracted much attention^3,5,10–12^. This is the central issue of a long standing debate in simple transition metal dichalcogenides which coexistence of CDW and superconductivity. The CDW is coupled to a periodic lattice distortion through a strong electron-phonon coupling. The transition is associated with a softening of a longitudinal acoustic phonon mode as temperature is lowered to *T*_CDW_^13^. It was already noticed that the high order phonon fluctuations and strong electron-phonon interactions explain some of the key features of the formation of the CDW in this system^14,15^.

Several studies have been performed in order to understand the vortex-pinning mechanism in more detail, that may led to real progress about the improvement of *J*_*c*_. The superconductors, main elementary interaction between vortices and pinning centers are magnetic interaction and the core interaction. The magnetic interaction arises from the interaction of surfaces between the superconducting and non-superconducting^6^ material parallel to applied magnetic field, which is usually in very small in the type II superconductors with very high Ginzburg-Landau parameter^16^. There are two predominant mechanisms of core pinning, *δT*_*c*_ associated with spatial fluctuation of the *T*_*c*_, and, charge carrier mean free path (*l*) near lattice defects are the main causes of *δl* pinning^7,17^. The strong pinning centers arises from defects and dopant atoms, which results in pinning by local variations in the mean free path^18^. Strong intrinsic pinning due to structural domains in the superconducting phase of 122 pnictides family observed, also field and temperature dependent *J*_*c*_ have been attributed to the inhomogeneous distribution of the dopant^19^.

The significant of the pressure can induce the enrichment of *T*_*c*_, hydrostatic pressure have extra benefits that are essential to the flux pinning. Pressure continuously reduces the lattice constants and causes the shrinkage of unit cells, charitable enlargement to the decrease of anisotropy. It can fortunate to induce the point defects, and then it is known that the formation energy of point defects decreases with the application pressure^20–22^. Polycrystalline samples easily affect the low-angle grain boundaries to the application of pressure, which sacrificing surface pinning. Hence, a higher ratio of point pinning to surface pinning centres is expected to increase formation energy and migration of grain boundaries under pressure. We found significant enhancement of *T*_*c*_, superconducting volume fraction, *H*_*c2*_, *H*_*irr*_, and *J*_*c*_ as we increase pressure in all compositions. This study motivates to analyse the enhancement of flux pinning and *J*_*c*_ to compare with high *T*_*c*_, pnictides and other superconducting samples.

Fe-NbSe_2_ belongs to the family of transition metal dichalcogenide compounds exhibit the superconducting transition. Another important feature of NbSe_2_ is the existence of a variety of vortex matter related phenomena, such as a pronounced peak effect and various phase transitions and instabilities^23–25^. In this work, we have investigated the pressure effects on *T*_*c*_, *J*_*c*_, and the flux pinning mechanism in Fe_x_NbSe_2_ for the first time. Hydrostatic pressure can induce a transition from the regime where pinning is controlled by spatial variation in the critical transition temperature (*δT*_*c*_) to the regime controlled by spatial variation in the mean free path (*δℓ*). In addition, *T*_*c*_ and low field *J*_*c*_ are slightly enhanced, also the *J*_*c*_ drops more sharply at high fields than at pressure. The current work demonstrates that the pressure increases the anisotropy and reduces the coherence length, ensuing in weak interaction of the vortex cores with the pinning centers. To understand the mechanism of vortex pinning in this system, scaling analysis of normalized pinning force density (F_p_/F_pmax_) as a function of reduced field (H/H_irr_) was performed and presented in this work.

## Results and Discussions

**Figure 1:**
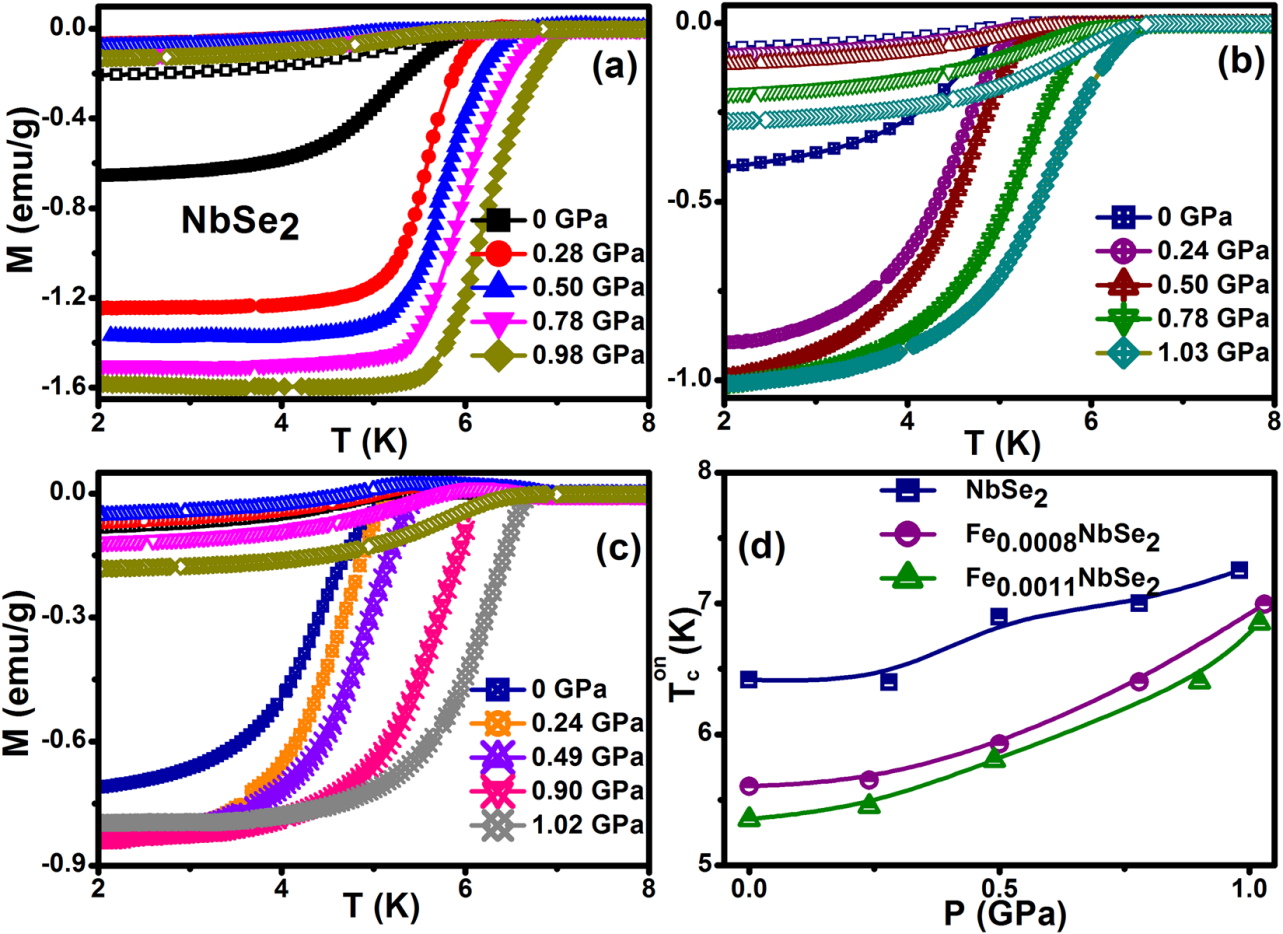
Temperature dependent zero-field cooling (ZFC) and field cooling (FC) magnetization measurements for (**a**) NbSe_2_ (**b**) Fe_0.0008_NbSe_2_ (**c**) Fe_0.0011_NbSe_2_ at constant magnetic field 20 Oe.

The temperature dependent magnetization curves for the zero-field cooling (ZFC) and field cooling (FC) curves at different applied pressure in the range 0–1 GPa for Fe_x_NbSe_2_ (x = 0, 0.0008 & 0.0011) are shown in Fig. 1(a–c). Pressure makes diminutive variation to the FC mode, representing that the strong flux pinning is engaged and shielding fraction was enhanced under pressure. The ambient superconducting critical temperatures (*T*_*c*_) of Fe_x_NbSe_2_ (x = 0, 0.0008 & 0.0011) are about 6.41, 5.60 and 5.35 K respectively and the application of continuous hydrostatic pressure increase of *T*_*c*_ are about 7.25, 6.99 and 6.85 K respectively at maximum pressure about ~1 GPa. The pressure enhances the *T*_*c*_ with the positive pressure coefficient (*dT*_*c*_*/dP*) with the rate are 0.86, 1.35 and 1.47 K/GPa and the results have been similar to the earlier literature^26–28^. The pressure evolution phase diagram for pure and Fe intercalated NbSe_2_ single crystals shown in the Fig. 1(d). The critical temperature was decreased due to the chemical pressure^3,5,7^ and it was increased due to the application of external hydrostatic pressure. The nature of relationship between the interlayer distance and *T*_*c*_ is a matter of fundamental importance in the understanding of the superconductivity of the layer compounds. To first approximation, the primary effect of pressure upon the physical properties of is regarded as being due to the reduction of interlayer distance. The Fermi level in NbSe_2_ is close to the maximum of the density of states *N*(*ε*_*F*_) of the narrow highly-hybridized subband, located between the completely full band formed by the *p*-state of Se, and the partially full band, formed by the *d* states of Nb^29,30^. If the charge transfer during intercalation shifts the Fermi level toward the sloped region of the density of states curve, then this can lead to a relatively big change in *N*(*ε*_*F*_) under pressure, and consequently, in *T*_*c*_. The last idea is supported by an increase of the width of the superconducting transition under pressure shown in the Fig. 1(d) and more pronounced manifestation of its stepped form. This type of evolution in the transition temperature to the superconducting state under high pressure, was also observed in cuprates^31^ and Fe based superconductors^32^ which also had a layered structure and a small anisotropy parameter.

**Figure 2:**
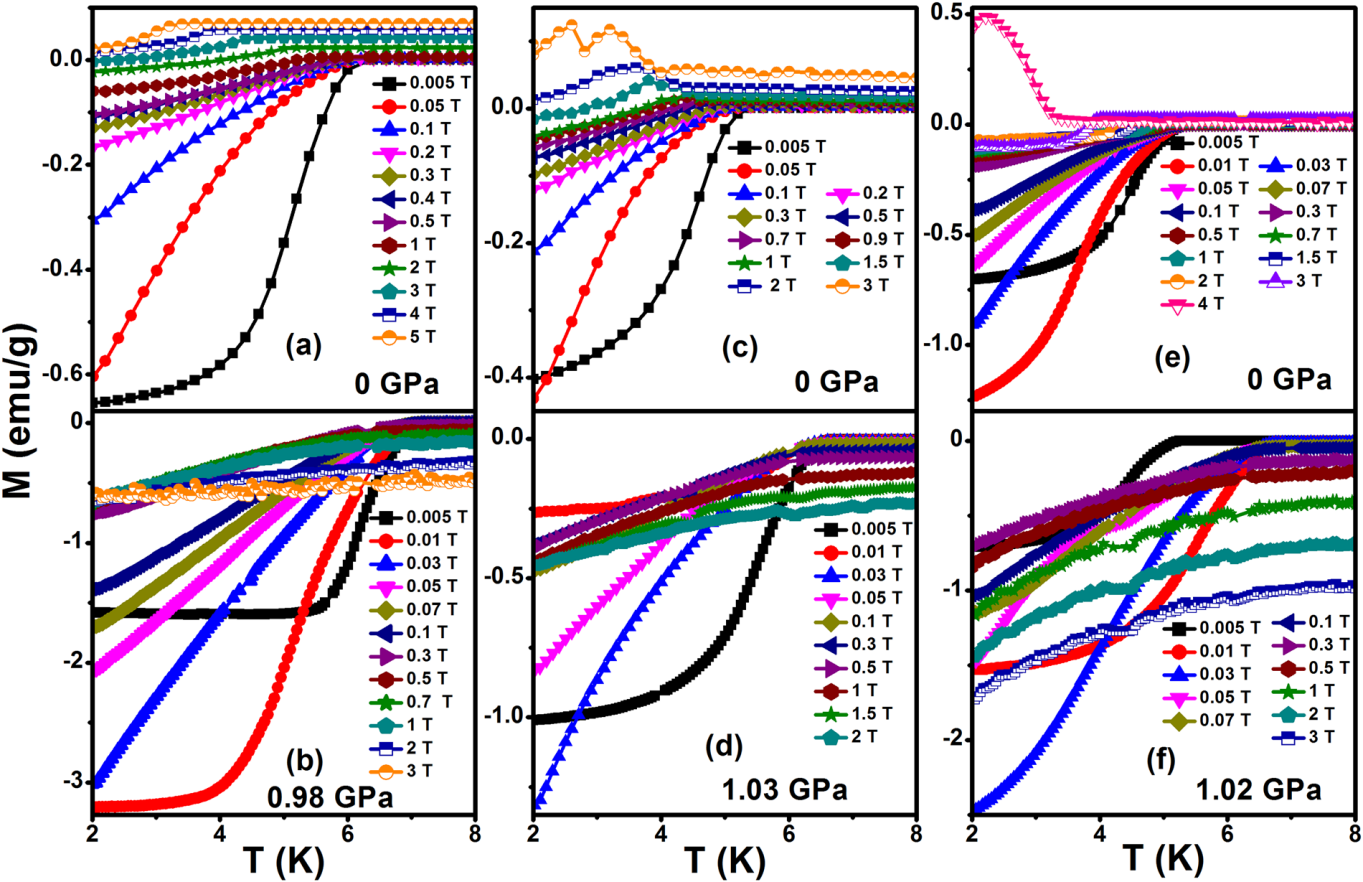
Temperature dependent magnetization measurements of the (**a** and **b**) NbSe_2_,(**c** and **d**) Fe_0.0008_NbSe_2_, (**e** and **f**) Fe_0.0011_NbSe_2_ at different magnetic field.

**Figure 3:**
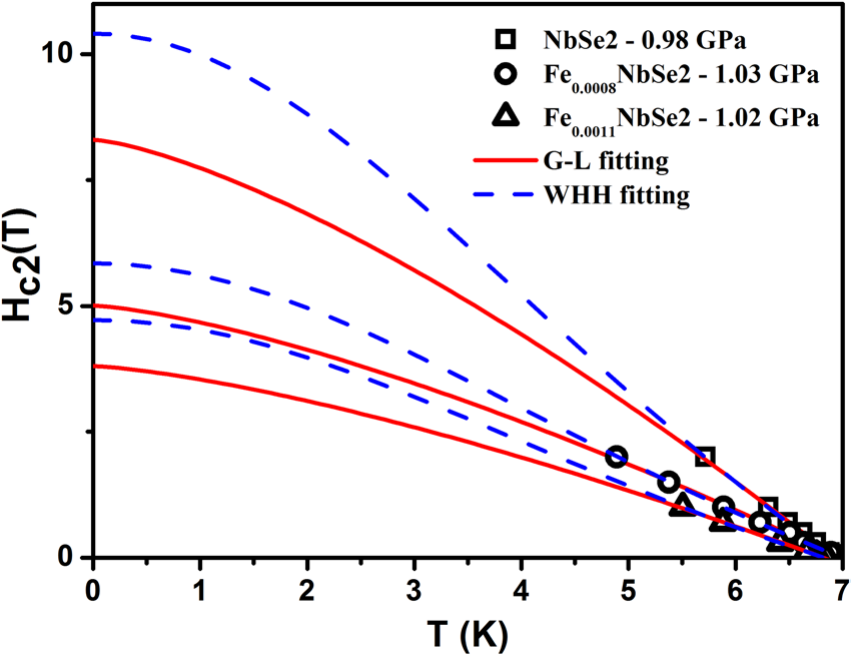
Temperature dependent upper critical field for the Fe_x_NbSe_2_ (x = 0.0008 & 0.0011). Solid lines are represent to fit the Ginzburg–Landau equation.

The *dc* magnetization measured for Fe_x_NbSe_2_ (x = 0, 0.0008 & 0.0011) with applied different magnetic field at maximum pressure ~1 GPa in the ZFC mode is shown in the Fig. 2. Figure 2 shows rapid decrease in the onset diamagnetic signal below *T*_*c*_ for Pure and Fe doped NbSe_2_. Figure 2 diamagnetic signal was suppresses due to the application of magnetic field at ambient pressure but field does not suppresses the signal at maximum pressure of ~1 GPa and it confirms that the pinning centers were increased due to application of pressure and will be briefly discussed later. The temperature dependent of upper critical field (*H*_*c2*_) for pure and Fe doped NbSe_2_ is shown in Fig. 3. Further, in a weak coupling case, the Pauli limited upper critical field is given by *H*_*P*_(0) = *1.84T*_*c*_, indicating that not only the orbital effect but also the Pauli spin paramagnetic effect (PSP) has an influence on the pair-breaking mechanism through the entire pressure region. The temperature dependence of critical field at absolute zero temperature *H*_*c2*_(0) can be determined by using the generalized Ginzburg-Landau model, ${H}_{c2}(t)={H}_{c2}(0){(1-{t}^{a})}^{b}$, where *t* = *T/T*_*c*_ is a reduced temperature with *a* = *1.39* and *b* = 1 associated with the large gaps that open in the Nb bands^33^ which gives the values slightly lower than that by WHH approach. According to the conventional single band Werthamer-Helfand-Hohenberg (WHH) theory, the orbital limited upper critical field (*H*^*orb*^_*c2*_) of type II superconductors, the ${H}_{c2}^{orb}(0)$ can be described, ${H}_{c2}^{orb}(0)=-0.693{T}_{c}{(d{H}_{c2}/dT)}_{Tc}$ for the dirty limit. The ${H}_{c2}^{orb}$ for pure and Fe doped NbSe_2_ are representatively plotted as the dashed lines in the Fig. 3 at ~1 GPa. The *H*_*c2*_ at 0 K and ambient pressure are 6.23 T (NbSe_2_), 2.72 T (Fe_0.0008_NbSe_2_) and 1.05 T (Fe_0.0011_NbSe_2_)^7^. From Fig. 3, ${H}_{c2}^{orb}(0)$ values calculated using the WHH equation of Fe_x_NbSe_2_ (x = 0, 0.0008 & 0.0011) are shown in the Table 1 for ambient and high pressure. The *H*_*c2*_(0) values are exposed that the Fe impurity destroys the superconducting properties by enhancing the pair breaking phenomena in the presence of magnetic field. An orbital and upper critical field values for all samples at pressure is higher than the ambient pressure, from the enhanced critical field implies that the pressure to induce the strong flux pinning granular for all samples. From this Ginzburg-Landau coherence length *ξ* = [*Φ*_0_*/2πH*_*c2*_(0)]^*1/2*^, where *Φ*_0_ = *2.07* *×* 10^*–7*^ *Gcm*^2^ the zero temperature coherence length *ξ*_*GL*_(0) for Fe_x_NbSe_2_ (x = 0, 0.0008 & 0.0011) are estimated and it is shown in the Table 1.

**Table 1:** Summary of the upper critical field for the Fe_x_NbSe_2_ (x = 0.0008 & 0.0011) single crystals.

Samples	*H*_*p*_(0) (*T*)	*H*_*c2*_(0) (*T*)	*ξ*_*GL*_(0) (*nm*)	*H*_*p*_(0) (*T*)	*H*_*c2*_^*orb*^(0) (*T*)	*H*_*c2*_(0) (*T*)	*ξ*_*GL*_(*0*) (*nm*)
	P = 0 GPa			P~1 GPa			
NbSe_2_	11.81	6.23	7.27	13.34	10.41	8.30	6.30
Fe_0.0008_NbSe_2_	10.31	2.72	11.01	12.87	5.84	5.01	8.11
Fe_0.0011_NbSe_2_	9.85	1.05	17.71	12.61	4.72	3.80	9.31

**Figure 4:**
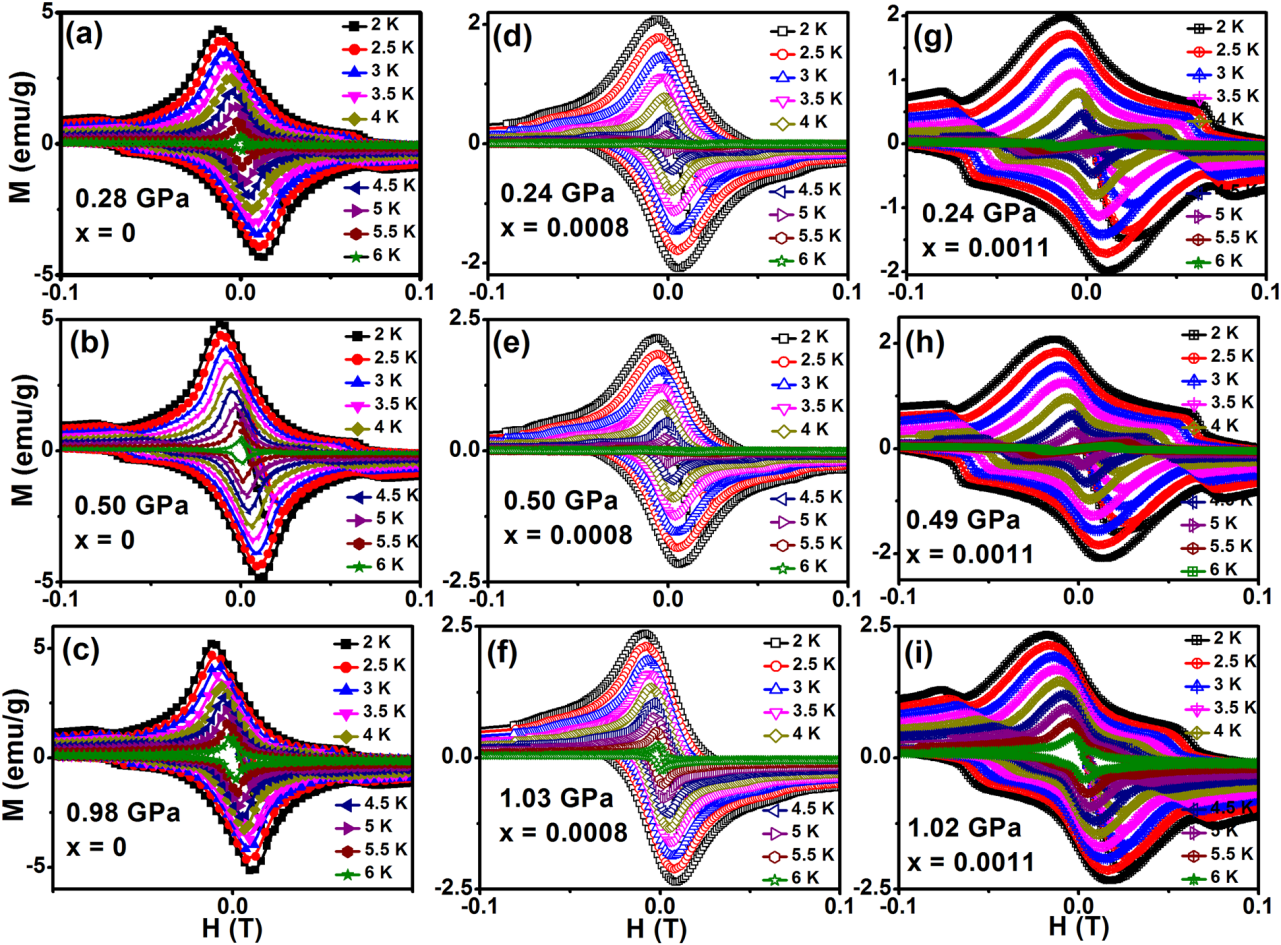
Field dependent isothermal magnetization curves of the (**a**–**c**) NbSe_2_, (**d**–**f**) Fe_0.0008_NbSe_2_ and (**g**–**i**) Fe_0.0011_NbSe_2_ at different temperatures for various hydrostatic pressures.

Figure 4 shows an isothermal field dependent magnetization curves for Fe_x_NbSe_2_ (x = 0, 0.0008 & 0.0011) at various temperature (2 to 5 K) under different hydrostatic pressures. The hysteresis loop clearly shows the type-II superconductivity and shows the second magnetization peak (SMP) effect which was obtained with constant sweeping rate *dH/dt* = 20 *Oe/s* for H on pure and Fe doped NbSe_2_ samples. One can clearly see that the superconducting magnetic hysteresis loop (MHL) arises from the flux gradient produced by the pinning of flux lines. Usually, flux jumps in superconductors occur in the low-field and low temperature regime due to the effect of thermomagnetic instability^34^. Significant progress for the understanding of the vortex phase diagram of superconductors with random quenched disorder and the nature of SMP has been made by considering an energy balance equation^35,36^. The SMP position *H*_*sp*_ moves up quickly as temperature decreases. The SMP phenomenon quite resembles that in YBa_2_Cu_3_O_7-δ_^31^ and Fe based superconductors^12,37–39^. At ambient pressure there is no SMP effect on the Fe_x_NbSe_2_ (x = 0, 0.0008 & 0.0011)^7^ but high pressure measurement observed SMP effect on all the samples due to the very low sweep rate of field. Pressure induces the SMP due to the decreases the interstitial distance Se-Nb-Se layer. Generally, field dependent critical current density *J*_*c*_ can be calculated from the MHL based on the Bean critical state model^40,41^ ${J}_{c}=20\Delta M/(b(1-b/3l))$, where $\Delta M(={M}_{\downarrow }(H)-{M}_{\uparrow }(H))$ is the width of the hysteresis loop (emu/cm^3^), *b* and *l* is the width and length of the samples (*b* < *l*) were measured in the cm.

**Figure 5:**
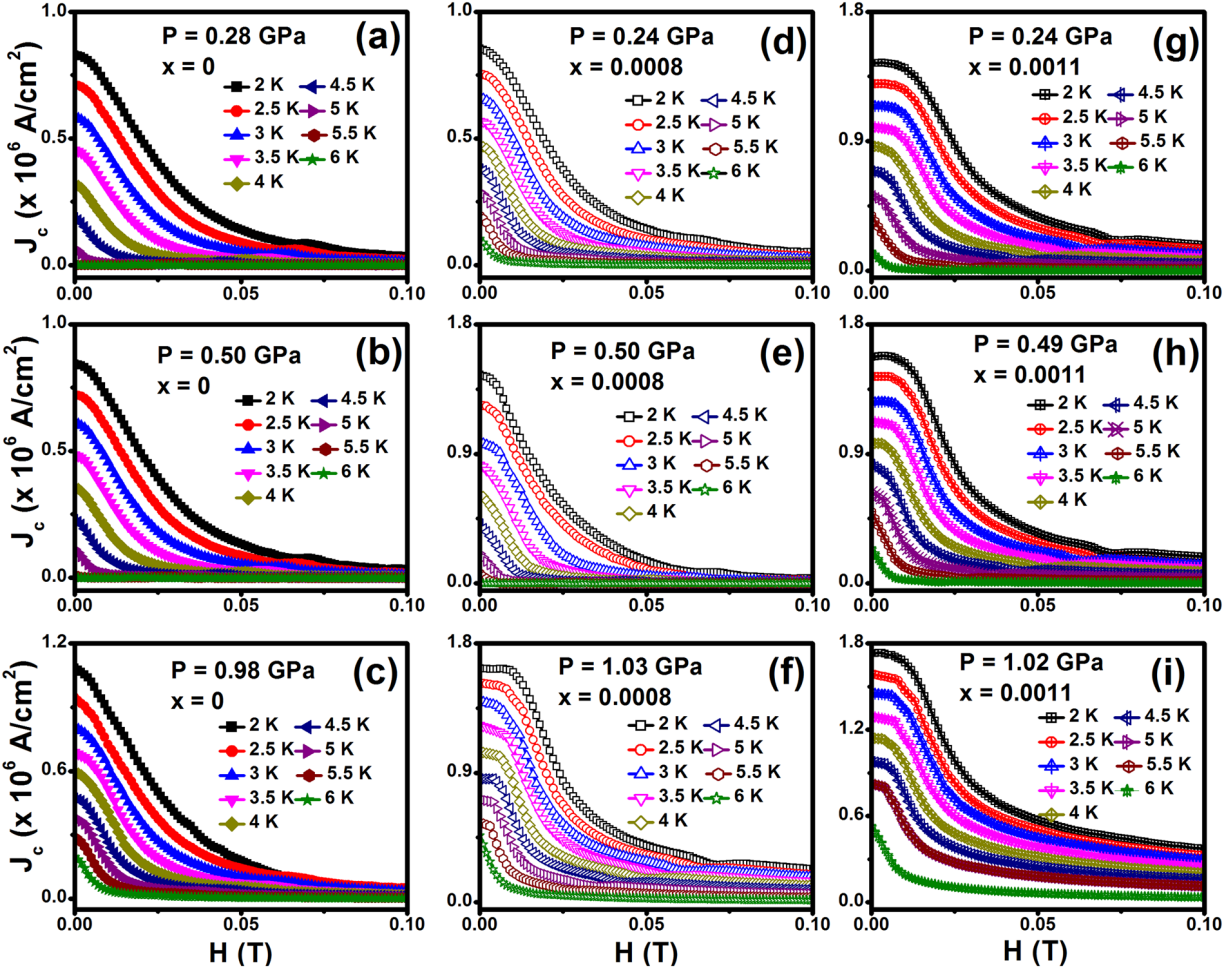
Field dependence of critical current density for (**a**–**c**) NbSe_2_, (**d**–**f**) Fe_0.0008_NbSe_2_ and (**g**–**i**) Fe_0.0011_NbSe_2_ at various hydrostatic pressures.

The magnetization isotherm curves have been measured under various pressures point out that the magnetic moment increasing with the application of hydrostatic pressure shown in the Fig. 4. From the magnetization curves have been taken at various *T*, we determined the temperature dependence of the magnetic field value corresponding to the second peak in magnetization, *H*_*peak*_. It follows that for the Fe_x_NbSe_2_ superconductor the position of the second-peak shifts toward lower fields monotonically on increasing the temperature, in analogy to what was observed in the YBa_2_Cu_3_O_7−δ_ superconductor^42^. Figure 5 shows the field dependence of *J*_*c*_ at different temperatures and various pressures for Fe_x_NbSe_2_ (x = 0, 0.0008 and 0.0011). We found that low field *J*_*c*_ was increased slightly under pressure. The *J*_*c*_ drops more quickly at higher fields, however compared to ambient pressure. The significant effect of pressure towards the enrichment of *J*_*c*_ could be noticeably seen. A pronounced second magnetization peak can be clearly observed. The second peak position *H*_*sp*_ moves up quickly as temperature decreases. Since the occurrence of SMP is quite advantageous in view of practical application and investigation of its origin is also helpful for understanding the fundamental question of underlying vortex physics, we performed a detailed vortex pinning study on this new layer superconductor. Magnetization relaxation in superconductors occurs because of the non-equilibrium spatial distribution of vortices which is determined by the competition of the external Lorentz force, the disorder induced pinning force density, and the thermal fluctuation.

The *J*_*c*_ value vs. reduced temperature (i.e. *1 − T/T*_*c*_) at 0 and 0.05 T under different pressures for Fe intercalated NbSe_2_. The data points in different fields and pressures follow a power law description [i.e. *J*_*c*_ *∝* (*1− T/T*_*c*_)^*β*^], where *β* is a critical exponent. At specific fields, Ginzburg-Landau theory predicts distinct vortex pinning mechanisms, with different values of exponent *β*. For example *β* = 1 corresponds to non-interacting vortices and *β* *≥* *1.5* corresponds to the core pinning mechanism. Our value of β~1.74 and 1.85 for zero field, and *β*~1.20 and 1.43 at 0.05 T, at 0 and 1.03 GPa, respectively, reveal a robust dependence of J_c_ on pressure. The low *β* values at high pressure show the weak field dependences of *J*_*c*_ in contrast to its values at low pressure. For type-II superconductors, attractive interactions between vortices and pinning centers prevent the movement of vortices.

To understand the origin pinning mechanisms in Fe_x_NbSe_2_ single crystals, the experimental results have been examined in the frame of collective pinning theory. Generally, core pinning comprises 1) *δl* pinning, which comes from spatial variation in the charge carrier mean free path *l*, and 2) *δT*_*c*_ pinning due to randomly distributed spatial variation in *T*_*c*_.

Griessen *et al*. approach:

1 $${{J}_{c}/{J}_{0}=\propto (1-{t}^{2})}^{5/2}{(1+{t}^{2})}^{-1/2}$$

**Figure 6:**
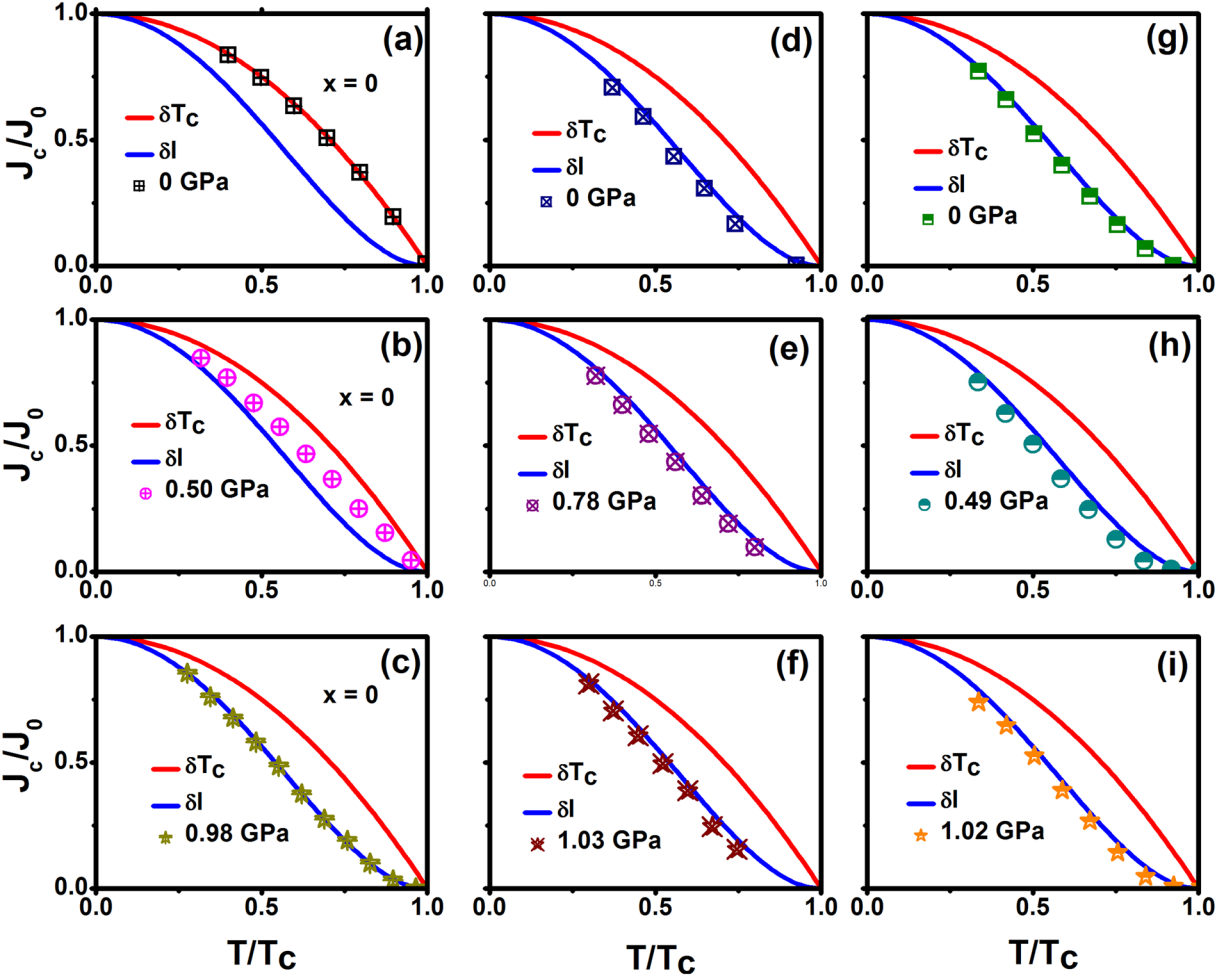
Reduced critical current density (J_c_/J_0_) as a function of reduced temperature (T/T_c_) for various pressure for the samples Fe_x_NbSe_2_ (x = 0, 0.0008 and 0.0011). Where (**a**,**b** and **c**) NbSe_2_, (**d**,**e** and **f**) are Fe_0.0008_NbSe_2_ and (**g**,**h** and **i**) Fe_0.0011_NbSe_2_. The red fitted line is δT_c_ pinning and the blue fitted line is δl pinning respectively.

Corresponds to *δl* pinning, while

2 $${{J}_{c}/{J}_{0}=\propto (1-{t}^{2})}^{7/6}{(1+{t}^{2})}^{5/6}$$

applies in the case of *δT*_*c*_ pinning, where *t* = *T/T*_*c*_. It should be noted that the flux pinning is two dimensional in such thin film, as the correlation length along the flux lines exceeds the film thickness. Figure 6 shows almost perfect overlapping of the experimentally obtained *J*_*c*_ values and the theoretically expected variation in the *δl* pinning mechanism at 0.05 T. This is in agreement with the observation of little change in *T*_*c*_ under high pressure.

**Figure 7:**
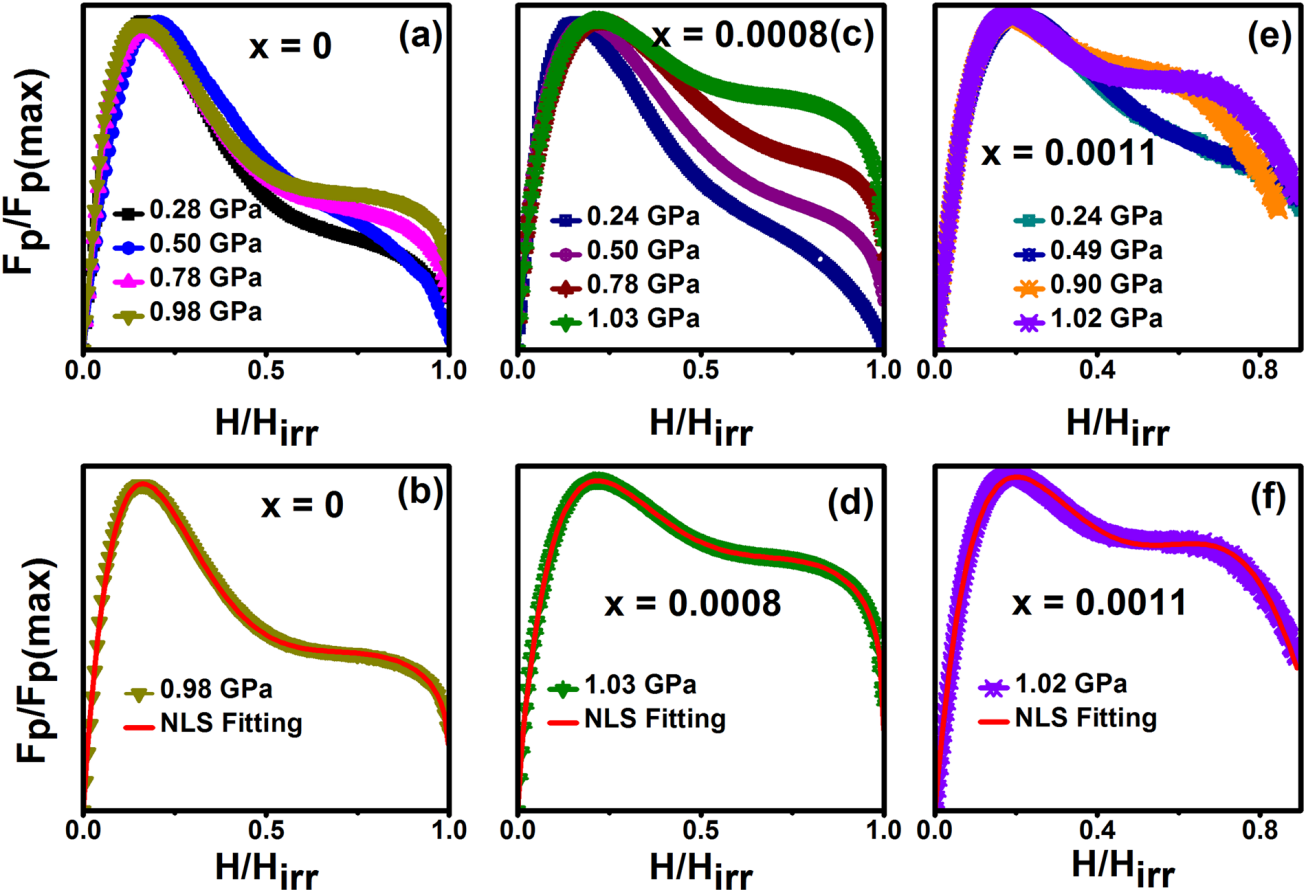
Normalized pinning force density (F_p_/F_p(max)_) as a function of reduced magnetic field (H/H_irr_) for various pressure for the samples Fe_x_NbSe_2_ (x = 0, 0.0008 and 0.0011). Where (**a** and **b**) NbSe_2_, (**c** and **d**) Fe_0.0008_NbSe_2_ and (**e** and **f**) Fe_0.0011_NbSe_2_. The solid lines are represents the nonlinear fitting using f_p_ = Ah^p^(1-h)^q^ + Bh^r^(1-h)^s^ at different pressures for the samples Fe_x_NbSe_2_ (x = 0, 0.0008 and 0.0011).

To understand *J*_*c*_ enhancement under pressure, the pinning force *F*_*p*_ = *J*_*c*_ *x H* is calculated and also to understand the vortex pinning mechanisms it is illustrative to plot the normalized pinning force density as function of applied magnetic field^43^. Shown in the Fig. 7 is the normalized pinning force, *f*_*p*_ = *F*_*p*_*/F*_*p*(*max*)_, plotted as a function of reduced field, *h* = *H/H*_*irr*_, at for various pressure for the samples Fe_x_NbSe_2_ (x = 0, 0.0008 and 0.0011). The temperature dependent irreversible field *H*_*irr*_ was determined using the Kramers plots present a wide linear behavior^44^. For the scaling, we can use the Dew-Hughes formula^43^, i.e., *f*_*p*_ = *h*^*p*^(*1-h*)^*q*^, where p and q are the parameters describing the pinning mechanism. In this model, *p* = *½* and *q* = 2 describes surface pinning while *p* = 1 and *q* = 2 describes point pinning as was predicted by Kramer^44^.The *H*_*irr*_ is estimated as the extrapolated zero *J*_*c*_^*0.5*^*H*^*0.25*^ *vs H* derived from the *J*_*c*_ isotherms. This, when fitted with Dew-Hughs function^43^, *h*^*p*^(*1-h*)^*q*^ results in *p~1.95* and *q*~*2.5* for Ru-doped crystals and the temperature independent *F*_*p*_ scaling and symmetric *F*_*p*_(*h*) curves with a peak at *h*_*max*_~*0.45* indicate a dense vortex pinning nanostructure. This could result from inhomogeneous distribution of Ru ions, which in turn produce a locally varying order parameter^19^. The field dependence of the reduced pinning force of NbSe_2_ and Fe doped NbSe_2_ explained clearly shows the point pinning with surface pinning mechanism at ambient pressure^7^.

The field dependence pinning force curves of NbSe_2_ at a temperature of 2 K at P~0.98 GPa are fitted with the Dew-Hughes point pinning model with *p* = *0.96* and *q* = *5.05* in addition to surface pinning with *r* = *0.56* and *s* = *1.71* in the form,

3 $${f}_{p}=A{h}^{p}{(1-h)}^{q}+B{h}^{r}{(1-h)}^{s}$$

Which is shown in the Fig. 7(b). The presence of surface pinning in the NbSe_2_ single crystal is due to the layered Se–Nb–Se structure with a larger value of *q* parameter reflecting the presence of dense point pinning centers^7^. The larger value of *q* parameter indication of the pinning center was increases due to the application external pressure for the NbSe_2_. The surface pinning mechanism was also induced by the application of pressure. The similar results have been observed for the Fe doped NbSe_2_ samples. The broadening of the pinning force with the application of pressure indicates that the pinning centers enhanced with the application of pressure for all the samples. Fe intercalated NbSe_2_ have more pinning centers compare between the pure NbSe_2_, it is due to the Fe ions incorporation of the Se-Nb-Se layers. For type II superconductors, vortices interact with pinning centers via the spatial variations of *T*_*c*_ (*δT*_*c*_ pinning) or by the scattering of charge carriers with mean free path *l* near defects (*δl* pinning). This implies that the leading pinning mechanism is core point pinning for pressures for all pure and Fe intercalated NbSe_2_ samples. From, observed results shown that the hydrostatic pressure has induced a perfect conversion from surface to point pinning.

In conclusion, we have examined systematically the flux pinning mechanism in optimally doped Fe_*x*_NbSe_2_ (*x* = 0, 0.0008, 0.0011) crystal under hydrostatic pressure and subsequently analyzed the *J*_*c*_ based on the collective theory. With the application of pressure upto ~1 GPa, *T*_*c*_ increases for all the samples. We have demonstrated that strong flux pinning in both low and high fields can be achieved by tuning the pinning force under pressure. Here we demonstrate that the pressure induce the transition *δT*_*c*_ to δl pinning mechanism in NbSe_2_, whereas the doped samples show δl pinning as the key pinning phenomena. This study also exhibits that the performance of Fe intercalated NbSe_2_superconductor in both low and high fields can also be further enhanced by pressure.

## Methods

The preparation method of single crystal of Fe_*x*_NbSe_2_ (*x* = 0, 0.0008 & 0.0011) is described elsewhere^7^. The compound crystallizes in hexagonal structure with space group *P6*_3_*/mmc*. Magnetization measurements at various pressures were performed using Physical Property Measurement System (PPMS, Quantum Design, USA). The external pressure was generated upto 1 GPa by a clamp type miniature hydrostatic pressure cell which is made of nonmagnetic Cu–Be alloy. The fluorinert FC#70 and FC#77 (1:1) mixture was used as a pressure transmitting medium and the *in-situ* pressure (P) was estimated from the superconducting transition of pure Sn as a manometer. Temperature dependence of magnetization M(T) was recorded upon zero field cooling at various pressures under external magnetic field of 20 Oe.

## Electronic supplementary material

Supplementary Information
