# Supplementary material for: Pressure assisted enhancement in superconducting properties of Fe substituted NbSe2 single crystal
Source: Sci Rep. 2018 Jan 19;8:1251. doi: 10.1038/s41598-018-19636-z (PMC5775345; doi:10.1038/s41598-018-19636-z)
Supplement: Supplementary file 1 — Supplementary Information [file 41598_2018_19636_MOESM1_ESM.pdf]

Pressure assisted enhancement in superconducting properties of Fe substituted $NbSe_2$ single crystal

Manikandan Krishnan[a], Rukshana Pervin[b], G. Kalai Selvan[a,c], Kannan Murugesan[a], L. Govindaraj[a], Akshay Kumar Verma[b], Parasharam M. Shirage[b] and S. Arumugam[a,*]

[a]Centre of High Pressure Research, School of Physics, Bharathidasan University, Tiruchirappalli 620024, India
[b]Discipline of Metallurgy Engineering and Materials Science & Physics, Indian Institute of Technology Indore, Simrol Campus, Khandwa road, Indore 453552, India.
[c]Department of Physics, University of Alabama at Birmingham, Birmingham, AL 35294, USA
[*]E-mail: sarumugam1963@yahoo.com

Structural analysis:

R. Pervin et al.,[1] reported and synthesis single crystals of Fe_xNbSe_2 (x = 0, 0.0008 & 0.0011) were prepared by the iodine vapour transport method. The phase purity and structural information determined from the XRD pattern of Fe_xNbSe_2 (x = 0, 0.0008 & 0.0011)[1].

Magnetization Measurement:

Measurement of pressure dependence of isotherm curves for Fe_xNbSe_2 (x = 0, 0.0008 & 0.0011) shown in the Fig. S1, Fig. S2 and Fig. S3 at different pressures ranges upto ~ 1 GPa using piston type pressure cell in the PPMS-VSM. From field dependent magnetization curves deduced the ΔM and from width of the MHL found to critical current density[2,3] of the Fe_xNbSe_2 (x = 0, 0.0008 & 0.0011) samples and also we have also measured the Meissner effect on this samples for different applied pressures.

We have been found that critical current density (J_c) was increased slightly under pressure. The J_c drops more quickly at higher magnetic fields[4], however compared to ambient pressure[1]. The effect of pressure towards the enhancement of J_c could be prominently seen in the Fig. S1, Fig. S2 and Fig. S3.

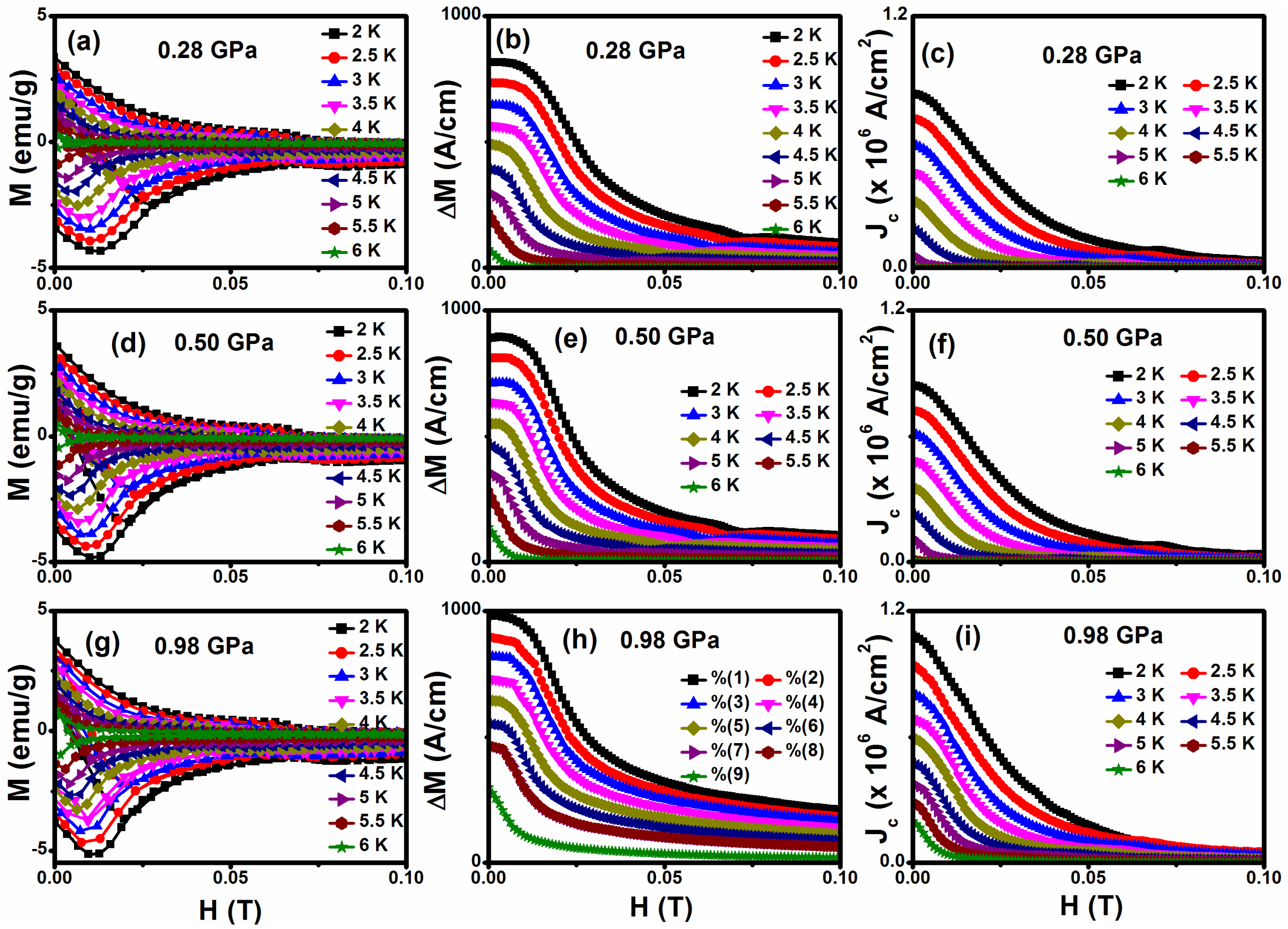

Fig. S1. *Field dependent isothermal magnetization curves of $NbSe_2$ and (a),(d) & (g), width of the MHL (b), (e) & (h) and critical current density (c), (f) & (i) $NbSe_2$ at different temperatures for various hydrostatic pressures.*

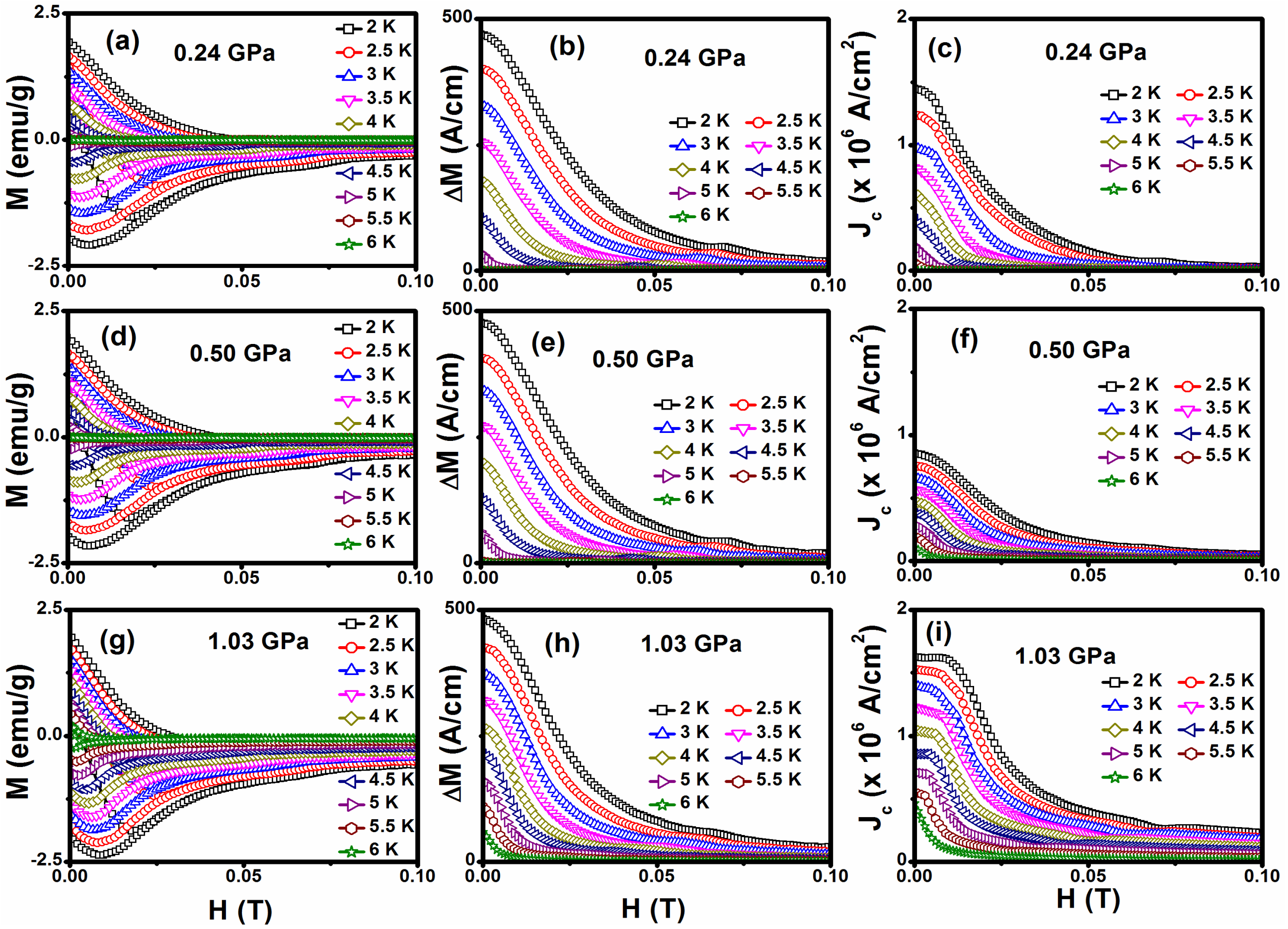

Fig. S2. *Field dependent isothermal magnetization curves of $NbSe_2$ and (a),(d) & (g), width of the MHL (b), (e) & (h) and critical current density (c), (f) & (i) $Fe_{0.0008}NbSe_2$ at different temperatures for various hydrostatic pressures.*

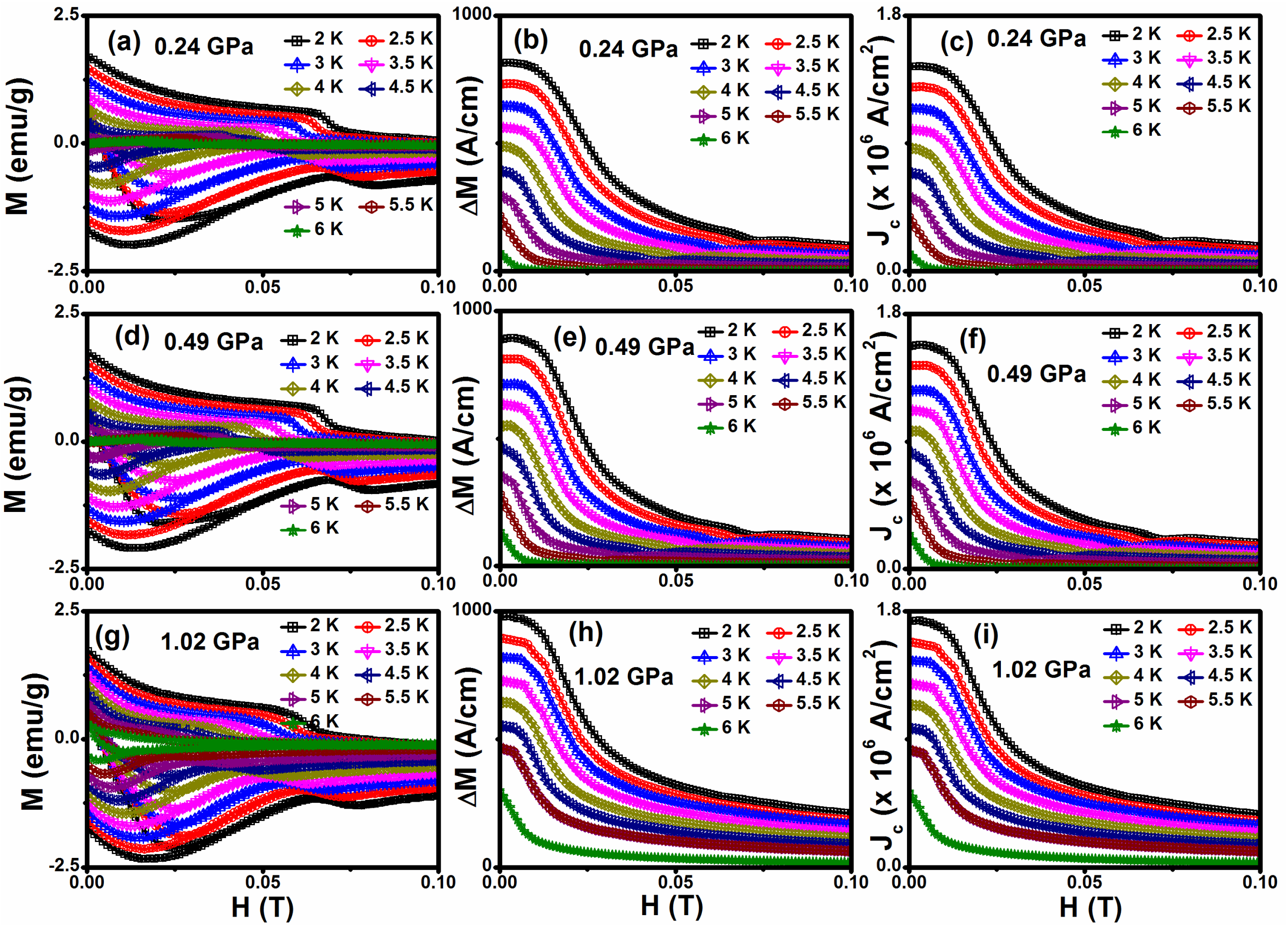

Fig. S3. Field dependent isothermal magnetization curves of $NbSe_2$ and (a),(d) & (g), width of the MHL (b), (e) & (h) and critical current density (c), (f) & (i) $Fe_{0.0011}NbSe_2$ at different temperatures for various hydrostatic pressures.

References:

1. Pervin, R. *et al.* Enhancement of superconducting critical current density by Fe impurity substitution in NbSe $_2$ single crystals and the vortex pinning mechanism. *Phys. Chem. Chem. Phys.* **19,** 11230–11238 (2017).

2. Bean, C. P. Magnetization of hard superconductors. *Phys. Rev. Lett.* **8,** 250–253 (1962).

3. Bean, C. P. Magnetization of high-field superconductors. *Rev. Mod. Phys.* **36,** 31–39 (1964).

4. Dewhughe.D. Flux pinning mechanisms in type-II superconductors. *Philos. Mag.* **30,** 293–305 (1974).
